# Reliability of Assessing Non-severe Elevation of Intracranial Pressure Using Optic Nerve Sheath Diameter and Transcranial Doppler Parameters

**DOI:** 10.3389/fneur.2019.01091

**Published:** 2019-10-22

**Authors:** Li-min Chen, Li-juan Wang, Lin Shi, Hong-xiu Chen, Xiao-han Jiang, Qian-qian Chen, Ying-qi Xing

**Affiliations:** ^1^Department of Neurology and Neuroscience Center, The First Hospital of Jilin University, Changchun, China; ^2^Department of Neurosurgery, The Affiliated Hospital of Changchun University of Traditional Chinese Medicine, Changchun, China

**Keywords:** intracranial pressure, transcranial Doppler, ultrasonography, optic nerve sheath diameter, non-invasive

## Abstract

**Background/Aims:** Non-invasive measurement of intracranial pressure (ICP) using ultrasound has garnered increasing attention. This study aimed to compare the reliability of ultrasonographic measurement of optic nerve sheath diameter (ONSD) and transcranial Doppler (TCD) in detecting potential ICP elevations.

**Methods:** Patients who needed lumbar puncture (LP) in the Department of Neurology were recruited from December 2016 to July 2017. The ONSD and TCD measurements were completed before LP.

**Results:** One hundred sixty-five participants (mean age, 41.96 ± 14.64 years; 80 men; 29 patients with elevated ICP) were included in this study. The mean ICP was 170 ± 52 mmH_2_O (range, 75–400 mmH_2_O). Univariate analyses revealed that ICP was non-significantly associated with TCD parameters and significantly associated with ONSD (*r* = 0.60, *P* < 0.001). The mean ONSD of the elevated ICP group was significantly higher than that of the normal ICP group (4.53 ± 0.40 mm vs. 3.97 ± 0.23 mm; *P* < 0.001). Multivariate linear regression determined that the difference between ICP and ONSD is significant.

**Conclusions:** In the early stage of intracranial hypertension, ONSD is more reliable for evaluating ICP than TCD.

## Introduction

Intracranial hypertension is an acute condition that impairs cerebral blood flow and metabolism, and it is associated with poor clinical outcomes and high mortality rates (1). Although the surgical placement of a transducer or lumbar puncture (LP) is the standard means by which clinicians can monitor intracranial pressure (ICP) (2), this procedure is associated with risks of cerebral damage and infection (3). Non-invasive alternatives for assessing ICP are, therefore, urgently needed. Changes in ICP depend on the pressure exerted by cranial contents [brain tissue, cerebrospinal fluid (CSF), and blood] on the wall of the cranial cavity. Recently, optic nerve sheath diameter (ONSD) has been proposed as a promising surrogate for the detection of elevated ICP with low intra- and inter-observer variability and high reliability (4–6). The fundamental basis for this method is the anatomical continuation of the optic nerve sheath across the three layers of meninges and its containment of CSF. International guidelines have recommended transcranial Doppler (TCD) as an effective and powerful method to confirm the clinical diagnosis of brain death (7). Brain death is the final manifestation of extremely elevated ICP. An increase in ICP beyond 40 mmHg (about 550 mmH_2_O) is considered a severe increase (8). However, the sensitivity of TCD in detecting non-severe elevation of ICP increases remains to be determined.

This study therefore compared the reliabilities of the measurement of ONSD and TCD parameters in detecting potential elevations of ICP.

## Materials and Methods

### Subjects

We performed a prospective observational study to compare the ICP estimated via measurement of TCD parameters and ONSD. Patients who needed LP in the Department of Neurology were recruited from December 2016 to July 2017. The experimental protocol was approved by the ethics committee of The First Hospital of Jilin University (approval number: 2016-376) and all participants provided written informed consent. Exclusion criteria were as follows: (1) age <18 years or >80 years; (2) cerebrovascular stenosis or deformity confirmed by imaging; (3) poor acoustic ultrasound windows; (4) cardiovascular diseases that cause hemodynamic variations and affect TCD readings, such as severe arrhythmia; and (5) suspected eye or orbit pathologies, such as glaucoma, lens opacity, or lens trauma. The data included daily recordings of age, sex, head circumference, waistline, body mass index (BMI), systolic blood pressure (SBP), diastolic blood pressure (DBP), and mean arterial blood pressure [MABP, (1/3 × SBP) + (2/3 × DBP)].

### Measurements

**Figure 1:**
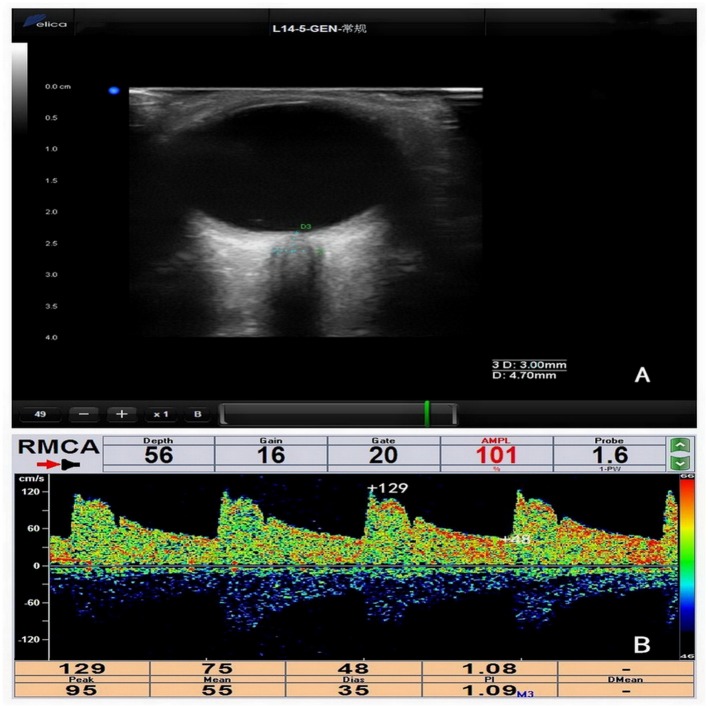
**(A)** Measurement of optic nerve sheath diameter (ONSD). ONSD was assessed 3 mm behind the orbit (ONSD = 4.70 mm). **(B)** The measurement of transcranial Doppler (TCD) parameters in the right middle cerebral artery (RMCA) (systolic blood flow velocity [TCD_vs_] = 129 cm/s, diastolic blood flow velocity [TCD_vd_] = 48 cm/s, mean blood flow velocity [TCD_vm_] = 75 cm/s, plasticity index [TCD_PI_] = 1.08).

DelicaMVU-6300 (Shenzhen, Guangzhou, China) was used to obtain both TCD and ONSD measurements: specifically, a 1.6 MHz probe and 14–5 MHz probe with B-mode, respectively, were used. The ONSD and TCD measurements were performed independently by two experienced observers who were blinded to each other's assessments, all of which were completed before LP (Figure 1). There were 5 min intervals between each of the TCD measurement, ONSD measurement, and LP. Every step of the operation adhered to the principle “as low as reasonably achievable” to avoid damage to the patients (9).

The patients were examined in supine position and ONSD was measured in both eyes. The probe was gently placed on the closed upper eyelid with standard ultrasound gel. We positioned the probe in a suitable angle to facilitate the display of the optic-nerve entry into the eyeball and froze the images. ONSD measurements were recorded at a depth of 3 mm behind the eye globe (4). Two measurements were obtained for each eye: one in the sagittal plane and the other in the transverse plane (4). The final results were derived from the mean value of each side.

The patients were examined in the supine and prone positions. The TCD measurements were performed over the acoustic bone windows for insonation of the left and right middle cerebral arteries (LMCA and RMCA, respectively) at a depth ranging from 50 to 65 mm; the left and right vertebral arteries (LVA and RVA, respectively), from 60 to 75 mm; and the basilar artery (BA), from 80 to 120 mm (10). The TCD probe was hand-held in place during the entire recording. Recorded TCD parameters included the following: systolic blood flow velocity (TCD_Vs_), diastolic blood flow velocity (TCD_Vd_), mean blood flow velocity (TCD_Vm_, [TCD_Vs_ + TCD_Vd_ × 2]/3), and plasticity index (TCD_PI_, [TCD_Vs_ – TCD_Vd_]/TCD_Vm_).

Thereafter, LP was immediately performed by a senior neurologist who was blinded to previous results according to a standard procedure. Patients were all in the left lateral position with knees and head maximally flexed. After a local skin disinfection and anesthesia with 5 ml of a 2% lidocaine hydrochloride solution, the LP needle was placed into the intervertebral space either between L3 (the third lumbar vertebrae) and L4 or between L4 and L5. Once CSF appeared, the pressure manometer was connected and the knees were extended to avoid falsely enlarged CSF pressure due to abdominal compression. Initial value of ICP was recorded. A pressure of >200 mmH_2_O was defined as elevated ICP (11).

### Statistical Analyses

Data were analyzed with SPSS software (version 20.0; Chicago, IL, USA). Continuous variables were expressed as means ± standard deviations (SDs) or medians with interquartile ranges (IQRs), and categorical variables were expressed as frequencies and percentages. Normality of the distribution was assessed by Kolmogorov–Smirnov test. We assessed differences in demographic variables, TCD parameters, and ONSD between the normal ICP group and elevated ICP group using independent samples *t*-test. Chi-squared test was used to compare proportions. Pearson correlation coefficient with a *P*-value was used to assess associations among ICP, TCD parameters, ONSD, and demographic variables, with *r* representing the correlation coefficient. Multiple linear regression models were constructed to identify the parameters that were significantly and independently associated with ICP. A receiver operating characteristic (ROC) curve was generated to determine the optimal cutoff point. All tests were two-tailed with significance set to *P* < 0.05.

## Results

**Figure 2:**
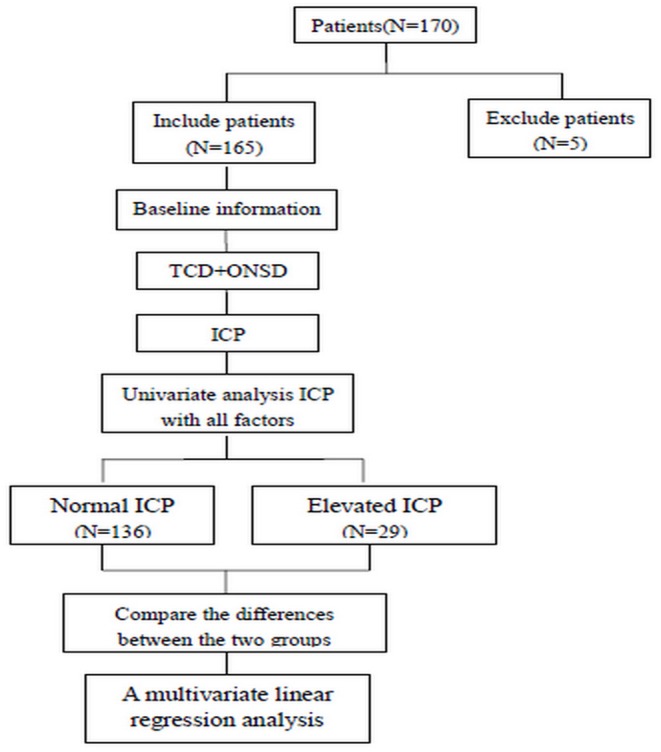
Flow chart of the experiment.

Among the 170 patients recruited in the present study, five were excluded: four owing to the lack of proper temporal windows for TCD examination and one owing to the lack of ONSD data (Figure 2). Thus, 165 participants (mean age, 41.96 ± 14.64 years; range, 18–77 years; men, 80; patients with elevated ICP, 29) were included in this study. Baseline data, TCD parameters, and ONSD values were available for each participant. The mean ICP was 170 ± 52 mmH_2_O (range, 75–400 mmH_2_O).

There were no significant differences in age, sex, head circumference, waistline, BMI, arterial blood pressure, or some TCD parameters (LMCA-TCDvs,vd,vm; RMCA-TCDvs,vd,vm; LVA-TCDvs,vd,vm,_PI_; RVA-TCDvs,vd,vm,_PI_; and BA-TCDvs,vd,vm,_PI_) between the normal and elevated ICP groups (Table 1).

**Table 1:** Demographic data in the normal and elevated ICP groups.

	**Normal (*n* = 136)**	**Elevated (*n* = 29)**	***P*-value**
Age, mean (SD), years	42.63 (14.85)	38.79 (13.44)	0.201
Male, (*n*, %)	64 (47%)	16 (55%)	0.540
Height, mean (SD), m	1.66 (0.08)	1.69 (0.08)	0.055
Weight, mean (SD), kg	63.42 (11.72)	68.98 (18.17)	0.124
BMI, mean (SD), kg/m^2^	22.95 (3.82)	23.88 (5.34)	0.385
Waistline, mean (SD), cm	79.52 (9.65)	84.14 (13.23)	0.084
Head circumference, mean (SD), cm	55.11 (1.83)	56.71 (2.29)	0.057
SABP, mean (SD), mmHg	128.97 (17.77)	135.97 (19.66)	0.085
DABP, mean (SD), mmHg	79.07 (11.30)	81.97 (14.08)	0.307
MABP, mean (SD), mmHg	95.71 (12.51)	99.97 (14.99)	0.162
ONSD, mean (SD), mm	3.97 (0.23)	4.53 (0.40)	**<0.001**
LMCA-TCD_vs_, mean (SD), cm/s	98.51 (20.07)	103.45 (22.22)	0.277
LMCA-TCD_vd_, mean (SD), cm/s	47 (11.52)	46.90 (12.97)	0.968
LMCA-TCD_vm_, mean (SD), cm/s	64.17 (14.03)	65.77 (15.72)	0.586
LMCA-TCD_PI_, mean (SD)	0.81 (0.12)	0.88 (0.14)	**0.008**
RMCA-TCD_vs_, mean (SD), cm/s	97.98 (20.04)	101.97 (22.20)	0.377
RMCA-TCD_vd_, mean (SD), cm/s	46.94 (12.00)	46.38 (12.14)	0.822
RMCA-TCD_vm_, mean (SD), cm/s	63.95 (14.33)	64.95 (15.16)	0.735
RMCA-TCD_PI_, mean (SD)	0.81 (0.12)	0.87 (0.12)	**0.019**
LVA-TCD_vs_, mean (SD), cm/s	48.94 (12.60)	48.38 (12.00)	0.831
LVA-TCD_vd_, mean (SD), cm/s	23.78 (6.81)	22.81 (7.42)	0.511
LVA-TCD_vm_, mean (SD), cm/s	32.17 (8.58)	31.33 (8.68)	0.651
LVA-TCD_PI_, mean (SD)	0.79 (0.11)	0.84 (0.17)	0.171
RVA-TCD_vs_, mean (SD), cm/s	48.13 (12.34)	44.81 (11.40)	0.187
RVA-TCD_vd_, mean (SD), cm/s	23.01 (6.05)	20.62 (5.89)	0.066
RVA-TCD_vm_, mean (SD), cm/s	31.38 (7.97)	28.68 (7.46)	0.112
RVA-TCD_PI_, mean (SD)	0.80 (0.12)	0.85 (0.17)	0.157
BA-TCD_vs_, mean (SD), cm/s	48.11 (12.47)	47.77 (14.69)	0.901
BA-TCD_vd_, mean (SD), cm/s	23.37 (6.50)	22.69 (7.69)	0.640
BA-TCD_vm_, mean (SD), cm/s	31.58 (8.31)	31.08 (9.75)	0.783
BA-TCD_PI_, mean (SD)	0.79 (0.10)	0.82 (0.15)	0.258
ICP, mean (SD), mmH_2_O	152 (28)	254 (58)	**<0.001**

*BMI, body mass index; SBP, systolic blood pressure; DBP, diastolic blood pressure; MABP, mean arterial blood pressure. [(1/3 × SBP) + (2/3 × DBP)]. Bold values are normal ICP, elevated ICP*.

**Figure 3:**
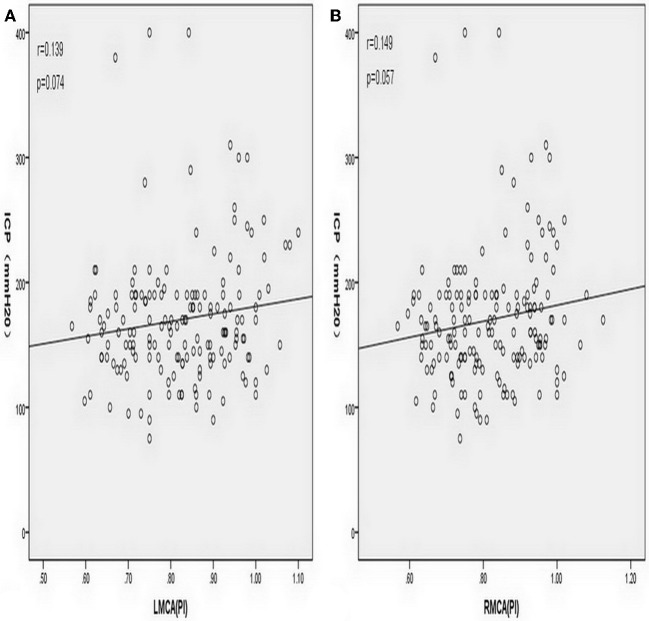
**(A)** Simple correlation analysis of all patients showed that intracranial pressure (ICP) was non-significantly associated with the plasticity index of the left middle cerebral artery (LMCA-TCD_PI_) (Pearson correlation coefficient *r* = 0.139, *P* = 0.074). **(B)** Simple correlation analysis of all patients showed that intracranial pressure (ICP) was non-significantly associated with the plasticity index of the right middle cerebral artery (RMCA-TCD_PI_) (Pearson correlation coefficient *r* = 0.149, *P* = 0.057).

**Figure 4:**
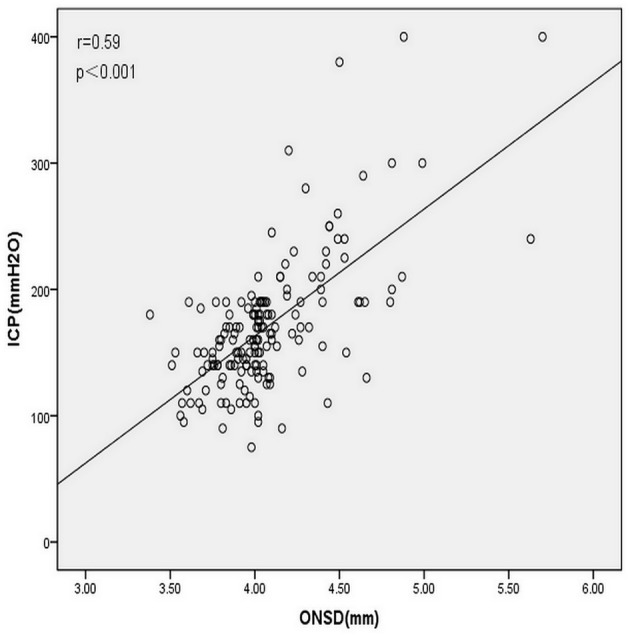
Correlation analysis of all patients revealed a significant correlation between intracranial pressure (ICP) and ultrasonographic measurement of ONSD (Pearson correlation coefficient *r* = 0.60, *P* < 0.001).

**Figure 5:**
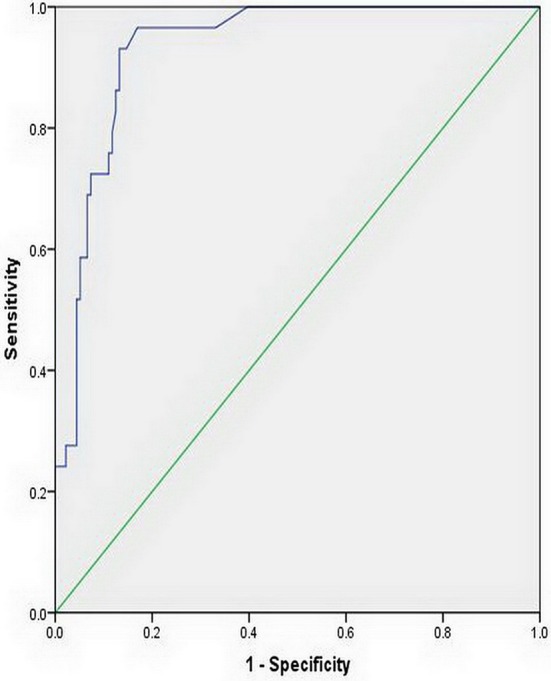
Receiver operating characteristic curve for the optic nerve sheath diameter. The area under the curve was 0.932 (95% CI: 0.893–0.971).

The LMCA-TCD_PI_ and RMCA-TCD_PI_ values were higher in the elevated ICP group than in the normal ICP group (0.88 ± 0.14 vs. 0.81 ± 0.12, *P* = 0.008 and 0.87 ± 0.12 vs. 0.81 ± 0.12, *P* = 0.019, respectively). In all patients, the mean LMCA-TCD_PI_ and RMCA-TCD_PI_ values were 0.82 ± 0.34 (range, 0.57–1.10) and 0.82 ± 0.12 (range, 0.57–1.13), respectively. However, the univariate analysis revealed that ICP was non-significantly associated with LMCA-TCD_PI_ (*r* = 0.139, *P* = 0.074; Figure 3A) and RMCA-TCD_PI_ (*r* = 0.149, *P* = 0.057; Figure 3B). The mean ONSD of all participants was 4.07 ± 0.34 mm (range, 3.38–5.70 mm) and that of the elevated ICP group was 4.53 ± 0.40 mm, which was significantly higher than that of the normal ICP group (3.97 ± 0.23 mm; *P* < 0.001). Univariate analysis revealed a significant correlation between ICP and ONSD in all the patients (*r* = 0.60, *P* < 0.001; Figure 4). In addition, ICP was also found to be significantly associated with SBP (*r* = 0.20, *P* = 0.006) and MABP (*r* = 0.18, *P* = 0.013) in all the patients. Multivariate linear regression analysis was performed to select the dependent variables, which include age, sex, MABP, LMCA-TCD_PI_, RMCA-TCD_PI_, and ONSD; SBP was not selected on account of its collinearity with MABP. Only ONSD and MABP remained in the final model: predicted ICP = 99.46 × ONSD + 0.66 × MABP −298.31. The Durbin–Watson value of the function was 1.69. Analysis of variance showed that the difference between ICP and ONSD is significant (F = 68.49, *P* < 0.001); the residuals were normally distributed, and the adjusted coefficient of determination (adjusted *R*^2^) was 0.451. Using the opening pressure of LP as the standard criterion, we generated an ROC curve to determine the cutoff point of ONSD (Figure 5). The ROC curve analysis revealed that the area under the curve (AUC) was 0.932 (95% CI: 0.893–0.971). The ONSD cutoff point for the identification of elevated opening pressure on LP was 4.14 mm, which yielded a sensitivity of 87% and a specificity of 93%.

## Discussion

Our results demonstrate that the LMCA-TCD_PI_ and RMCA-TCD_PI_ values of the elevated ICP group were higher than those of the normal ICP group. However, the univariate analysis showed that ICP is non-significantly associated with LMCA-TCD_PI_ and RMCA-TCD_PI_. The mean ONSD of the elevated ICP group was significantly higher than that of the normal ICP group. The univariate analysis of all patients revealed a significant correlation between ICP and ONSD. The multivariate linear regression analysis determined that the difference between ICP and ONSD is significant.

Similar to the results of a previous study, results of the current study confirm that ONSD is correlated with ICP (12). Ultrasound measurement of ONSD is a non-invasive, low-cost, quick bedside operation. Measurements of ONSD have demonstrated additional potential for the detection of increased ICP when combined with computed tomography or magnetic resonance imaging; the results obtained using these methods were consistent with those of ultrasound imaging (13, 14). Studies on Western populations indicate 5.0–5.9 mm as a reliable cutoff value to predict an ICP of >200 mmH_2_O (5, 15–17); however, a study in Bangladesh showed that an ONSD of >4.75 mm was considered abnormal (18). In our study too, the ONSD cutoff point for the identification of an elevated opening pressure of LP was <5.0 mm. These differences in cutoff points may implicate genetic differences (19). Alternatively, the patients in previous studies had severe injuries or required treatment in the intensive care unit; interventions such as intensive care or intubation may have affected the results. In contrast, we measured ONSD and ICP at an early stage of high ICP without interference from critical care measures.

After its introduction in 1982, TCD was quickly applied to study the intracranial blood flow patterns in cases of increased ICP and cerebral circulatory arrest (20). International guidelines have recommended TCD as an effective and powerful method to confirm the clinical diagnosis of brain death (7). Low-frequency transducers (<2 MHz) placed on the scalps of patients over specific acoustic windows have hitherto facilitated the display of intracranial arterial vessels and evaluation of the cerebral blood flow velocity as well as its changes across various conditions. However, while some studies have suggested that TCD can be used to evaluate ICP (21), others have found that it is unreliable (22). Moreno et al. reported that PI = 0.48 + 0.03 ICP [*r*^2^ = 0.69, *P* < 0.000; (21)] in their study, and the mean ICP and PI values were 22.07 ± 17.29 mmHg (~300 ± 235 mmH_2_O) and 1.26 ± 0.73, respectively. Conversely, Robba et al. found that PI did not significantly correlate with ICP (22), and Zweifel et al. reported that the correlation between PI and ICP was 0.31 (*P* < 0.001) (23); these results indicate a diminished value of PI for the assessment of ICP. The present investigation also found that PI-based predictions of the early stages of high ICP may not be as accurate as expected; we observed the mean ICP and PI to be 170 ± 52 mmH_2_O and 0.82 ± 0.34, respectively, and a non-significant correlation between ICP and PI. These results indicate that PI may not be suitable for the evaluation of the early stages of high ICP; however, it may be helpful when applied to cases of significantly elevated ICP and even extremely elevated ICP to diagnose brain death (20). Previously, researchers have mostly used MCA when using TCD to evaluate ICP. Nevertheless, no study has hitherto proven the feasibility of evaluating ICP using VA and BA. Our study shows that there are no significant differences in the parameters of LVA, RVA, or BA between the normal and elevated ICP groups. Some researchers have recently explored the use of other hemodynamic parameters derived from TCD to evaluate ICP. Ragauskas et al. described the two-depth high-resolution TCD insonation of the ophthalmic artery with a pressure cuff surrounding eye-ball tissues (24). This method does not require calibration, and it is performed when the blood flow parameters of the intracranial and extracranial ophthalmic artery segments are approximately equivalent and the external pressure equals the ICP. Schmidt et al. constructed a “black-box” model based on the arterial blood pressure and the TCD waveform of arterial blood flow, which demonstrates the promise of reliability in predicting ICP (25). Furthermore, TCD measurements of veins may reportedly have predictive value in the evaluation of ICP (22). However, these methods require experienced operators. Hence, the applications of these techniques to clinical practice are currently limited, and research of other hemodynamic parameters is thus warranted.

## Limitations

The present study was conducted in a single center. A multi-center study with a larger sample size is, therefore, required. While this investigation focused on non-invasive methods for the assessment of early-stage high ICP, further research should apply the same methods to assess extremely high ICP. Third, the individual ONSD differences should also be considered in future research.

## Conclusion

Our results indicate that in the early stage of intracranial hypertension, cerebral arterial hemodynamics assessed by TCD could not predict ICP with sufficient reliability; however, ONSD may be a reliable predictive index for elevated ICP.

## Data Availability Statement

The datasets generated for this study are available on request to the corresponding author.

## Ethics Statement

The studies involving human participants were reviewed and approved by The First Hospital of Jilin University (approval number: 2016–376). The patients/participants provided their written informed consent to participate in this study.

## Author Contributions

YX and LW: conceived and designed the experiments. LC, LS, HC, XJ, and QC: performed the experiments. LC: analyzed the data. LC and LW: wrote the paper.

### Conflict of Interest

The authors declare that the research was conducted in the absence of any commercial or financial relationships that could be construed as a potential conflict of interest.
